# Supplementary material for: Are carbon emissions trading and green financial instruments synergistic? -Comprehensive quantitative research based on content analysis
Source: PLoS One. 2024 Mar 7;19(3):e0298601. doi: 10.1371/journal.pone.0298601 (PMC10919601; doi:10.1371/journal.pone.0298601)
Supplement: S1 Data — (ZIP) [file pone.0298601.s001.zip › Data/地级市Z_F工作报告.zip!/┴╔─■/╙¬┐┌╩╨/└┤╫╘╣1⁄2╓┌║┼í╛┬φ┐╦╩2╛▌═°í┐_2011.docx]

各位代表：

　　现在，我代表市政府向大会报告工作，请予审议，并请市政协委员和列席会议的同志提出意见和建议。

　　一、“十一五”时期的回顾和2010年工作

　　“十一五”时期，是营口发展史上极不平凡的五年。面对复杂多变的宏观经济环境和艰巨繁重的发展改革任务，在省委、省政府和市委的正确领导下，在市人大、市政协的监督支持下，市政府带领和依靠全市人民，深入贯彻落实科学发展观，团结奋斗，埋头苦干，圆满完成了“十一五”规划确定的主要目标和任务，谱写了振兴发展的新篇章。

　　“十一五”时期，全市生产总值年均增长20%，去年达到1002亿元，5年增长1.6倍。地方财政一般预算收入年均增长44%，去年达到100.1亿元，5年增长5.3倍。全社会固定资产投资年均增长35%，累计达到3100亿元，比“十五”时期增长5.3倍。社会消费品零售总额年均增长18.5%，去年达到250亿元，5年增长1.3倍。实际利用外商直接投资年均增长28%，累计达到19亿美元，比“十五”时期增长2.4倍。外贸出口总额年均增长17%，累计达到76亿美元，比“十五”时期增长1.3倍。城镇居民人均可支配收入年均增长11.7%，去年达到17760元，5年增长1倍；农村居民人均纯收入年均增长11.6%，去年达到8600元，5年增长1倍。主要经济指标增长速度位居全省前列。

　　“十一五”时期，营口发展实现了历史性的跨越。这五年是经济实力提升最快、社会建设成效最好的五年，也是城乡面貌变化最大、人民群众得到实惠最多的五年。

　　“十一五”时期，营口不断深化市情认识，优化区域布局，形成了沿海经济发展的新格局。这是贯彻落实省委、省政府重大决策的具体行动，也是着眼未来作出的战略选择。特别是随着辽宁沿海经济带开发开放上升为国家战略和沈阳经济区被确定为综合配套改革试验区，营口沿海经济带开发建设步伐不断加快，成为全市加速发展的新引擎。

　　“十一五”时期，营口坚持以经济建设为中心，在原来工作的基础上，经济发展整体水平大幅提升。经济总量连续4年居全省第4位，增长速度连续5年居全省第1位。营口的知名度越来越高，社会关注度越来越大，已成为辽宁新的重要增长区域。

　　“十一五”时期，营口坚持从实际出发，加强经济社会发展薄弱环节，形成了全面协调可持续发展的良好局面。新农村建设扎实推进，区域发展协调性增强。教育、文化发展迈上新台阶，卫生、体育事业取得新成绩。就业规模日益扩大，社会保障体系建设进一步加强。能源资源节约和生态环境保护取得新进展。沿海特大城市建设框架全面拉开，一个充满生机和活力的新营口正在形成。

　　各位代表：刚刚过去的2010年，经过全市上下的共同努力，全面完成了市十四届人大三次会议确定的目标和任务，不仅实现了“十一五”规划的圆满收官，也为“十二五”发展打下了良好基础。

　　预计2010年，全市生产总值增长18%，地方财政一般预算收入增长45%，全社会固定资产投资增长31%，社会消费品零售总额增长18.5%，实际利用外商直接投资增长70%，外贸出口总额增长83%，城镇居民人均可支配收入增长12%，农村居民人均纯收入增长13%。居民消费价格总水平上涨2.9%，低于全省平均水平。

　　（一）招商引资开创新局面。把招商引资上项目作为经济工作的重中之重来抓，在全市掀起了招商引资的新高潮。通过落实招商责任，强化招商队伍，完善招商机制，优化招商环境，招商引资取得明显成效。伊顿电器、信义玻璃等一批重大项目签约；可口可乐、环渤海钢材城等一批规模大、辐射力强的项目相继开工建设。全市开工建设千万元以上项目1057个，其中亿元以上项目397个。新批外商投资项目85个，其中投资千万美元以上项目57个。

　　（二）工业结构调整迈出新步伐。规模以上工业增加值实现610亿元，增长22%。冶金、石化、装备制造、镁质材料、纺织服装和新型建材等六大主导产业产值均突破百亿元，实现工业增加值477亿元，占全市工业增加值的78.2%。新材料、新能源等新兴产业发展势头强劲。全国首家低碳生态科技产业园落户产业基地，新东北电气、东方光大等新兴产业项目加快推进。汽保、乐器两大特色产业园建设步伐加快，在全国率先建立了汽车保修设备公共研发服务平台。科技创新能力进一步增强，引进海外研发团队10个，并购国内外企业7家。品牌战略有效实施，新增中国驰名商标7件。高新技术产品增加值实现110亿元，增长35%。民营经济增加值实现480亿元，增长28%。

　　（三）县域经济和城区经济发展迈上新台阶。农业生产克服多种自然灾害，粮食产量67.5万吨，蔬菜产量85万吨，水果产量68万吨，水产品产量53万吨，肉蛋奶产量35万吨。新发展农事企业73家。新增农民专业合作社291个，创建省级示范社16个。新建设施农业小区449个、2.5万亩。成功举办第二届农博会，新创省以上农产品名牌16个。农产品加工业产值实现198亿元，增长30%。超额完成了县域经济“三年倍增”计划。县域生产总值增长25%，地方财政一般预算收入增长44%。大石桥市在全国百强县排名由76位升至61位，盖州市在全省县域经济排名由29位升至17位。省委、省政府在我市召开了城区经济现场会和温泉旅游现场会，鲅鱼圈经验在全省得到推广。提前一年完成城区经济“三年倍增”计划。城区生产总值增长36.8%，地方财政一般预算收入增长88.5%。服务业呈现大发展、快发展的良好态势。服务业增加值实现370亿元，增长19%。旅游总收入116亿元，增长30%。金融业发展迅猛，招商、沿海银行实现开业，银行业金融机构存款余额和域内外银行贷款余额均突破1000亿元。房地产市场购销两旺，保利、恒大、嘉里、佳兆业等一批知名房地产企业进入营口。房地产销售额162亿元，增长60%。

　　（四）沿海经济带开发建设进入新阶段。全年沿海经济带开发土地53平方公里，完成投资700亿元。营口港货物吞吐量达到2.25亿吨，增长28.3%；集装箱运量达到333.8万标箱，增长31.6%。开发区南部新区建设全面展开，船舶产业基地和望海珍珠湾开工建设，开工建设亿元以上项目97个。北海新区建设全面启动，编制了城市战略和产业发展规划，路网、回迁楼和盖州市行政中心等一批项目开工建设；规划建设了12平方公里的电机产业园，洽谈和引进电机产业项目205个，其中开工建设101个。产业基地项目引进取得新进展，开工建设千万元以上项目100个。高新区晋升为国家级高新技术产业开发区，辽宁渤海科技城建设全面展开，开工建设千万元以上项目60个。仙人岛能源化工区白沙湾黄金海岸正式开园，世博“伦敦零碳馆”落户仙人岛，开工建设千万元以上项目38个。中小企业园一期工程全面完成，开工建设各类项目119个，一期南扩工程启动实施。大石桥沿海新兴产业区开发建设取得新成绩，开工建设千万元以上项目132个。

　　（五）基础设施和生态环境建设实现新突破。为营口市区提供水源的石门水库供水工程完工，大伙房水库输水配套工程一期完成主体工程，主城区基本实现全天候供水。辽河特大桥建成通车。营口机场前期工程开工建设，城际铁路纳入全省高铁建设规划。城市“东扩、西改、南进、北转”全面推进，站前区、西市区城市改造步伐加快，营东新城建设取得较大进展。对老城区31条主次干道和53条街巷路进行了改造。投资16.3亿元，完成造林20.6万亩，新增城市绿地0.5万亩。投资17.8亿元，完成了一批农田水利工程。重点河流治理步伐加快，民兴河治理基本完成。矿山整治效果明显。东部污水处理厂正式运行，24项重点工业节能减排和资源综合利用项目全部完工，实现了“十一五”节能减排目标。

　　（六）民生工程实施取得新成效。全面完成15件重点民生工程。投资6.8亿元，实施了我市历史上规模最大的集中供热改造工程，做到了当年开工、当年供热。对108个旧住宅小区进行了综合改造。解决了农村14万人饮水安全问题。完成农村改厕1万座。新建和改建学校48所。新增实名制就业6.75万人，保持零就业家庭动态为零，城镇登记失业率控制在3.4%，低于全省平均水平。提高了企业退休职工养老金、城乡居民低保、在乡复员军人生活补助、农村五保供养和新农合及城镇居民医保补助标准。“定点帮扶”和“千企百村对接帮扶活动”取得实效，投入扶贫开发资金4000多万元。新建廉租房3.2万平方米、经济适用房43.7万平方米，棚户区改造125万平方米。

　　（七）社会事业发展取得新进展。各级各类教育水平不断提高，基础教育在全省率先实现全域高水平、高质量发展。文化事业实现新突破，文化体制改革试点经验在全国推广，文艺表演团体转企改制成为文化部10个国家文化创新工程项目之一。农家书屋覆盖到每个村，数字电影覆盖到每个乡镇。

　　医疗卫生水平进一步提高，医药卫生体制综合改革试点工作启动实施，15家基层医疗卫生服务机构实施了国家基本药物制度。人口和计划生育服务能力进一步增强，人口自然增长率1.27％。。群众体育和竞技体育蓬勃发展，我市在省十一届运动会上获得好成绩。社区建设进一步加强。创建全国文明城市工作有了新的进展，在全省参与全国城市公共文明指数测评的5个城市中我市列第2位。

　　（八）民主法制建设取得新进步。认真落实市人民代表大会及其常委会的决议决定，自觉接受依法监督。认真听取市政协和民主党派、工商联、无党派代表人士的意见和建议，自觉接受民主监督。办理省、市人大代表建议218件，省、市政协提案209件，办复率和满意率均为100%。“五五”普法工作任务全面完成，依法治市工作取得实效。行政管理体制改革进一步深化，政务公开、行政监察、审计监督力度不断加大。认真受理群众来信来访和投诉，信访办结率达到95%以上，群众投诉办结率达到85%以上。

　　狠抓社会稳定、安全生产和食品药品安全工作。新闻出版、双拥共建、民兵预备役、民族宗教、人防、史志、档案、气象、地震等工作不断加强，老龄、慈善、残疾人、妇女儿童等各项事业取得了新成绩。

　　各位代表：营口的变化令人瞩目，营口的发展振奋人心。这些成就的取得，是省委、省政府和市委正确领导的结果，是市人大、市政协监督支持的结果，是全市广大干部群众共同努力的结果。在此，我代表市政府，向辛勤工作在各条战线的全市人民，向人大代表、政协委员，向各民主党派、工商联、无党派人士和人民团体，向中省直单位、驻营部队、武警官兵和公安民警，向所有关心、支持营口发展的各界人士表示衷心的感谢和崇高的敬意！

　　在总结回顾过去工作的同时，也清醒地看到，当前营口经济社会发展还面临一些困难和问题。主要是：经济总量还不大，并且缺少对全市经济发展有牵动和支撑作用的大项目；产业集群发展滞后，自主创新能力弱，服务业规模小、层次低；城乡规划建设和管理水平不高，城市功能不完善，城乡面貌有待进一步改观；城乡居民收入水平提高不快，社会保障能力不强，一些群众生活仍然比较困难；生态环境治理任务重，节能减排压力大；政府行政效能亟待提高，政务环境有待进一步改善。对此，市政府将在今后的工作中，采取有力措施，认真加以解决。

　　二、“十二五”时期发展目标和主要任务

　　各位代表：“十二五”时期，是营口全面建设小康社会的关键时期，也是营口实现全面振兴的重要战略机遇期。市政府将倍加珍惜难得的发展机遇，倍加珍惜来之不易的大好形势，更加积极主动地做好各项工作，不断开创营口经济社会发展的新局面。

　　按照省委、省政府和市委的部署，市政府在认真听取各方面意见的基础上，编制了《营口市国民经济和社会发展第十二个五年规划纲要（草案）》，提请本次会议审议。

　　“十二五”时期，全市经济社会发展的指导思想是：高举中国特色社会主义伟大旗帜，以邓小平理论和“三个代表”重要思想为指导，深入贯彻落实科学发展观，顺应全市人民过上更好生活新期待，紧紧抓住中央进一步实施东北地区等老工业基地振兴、辽宁沿海经济带开发开放上升为国家战略、沈阳经济区被确定为综合配套改革试验区的难得机遇，以建设沿海经济强市为目标，以产业升级、城市转型和民生改善为重点任务，坚持“以港兴市、产业立市、文化强市”，加快转变经济发展方式，推进改革开放和自主创新，完善社会建设和生态环境保护，把营口建设成为具有较强竞争力的新型产业基地、东北物流枢纽和现代化港口特大城市，努力实现新的跨越和率先崛起，为提前全面建成小康社会奠定更加牢固的基础。

　　“十二五”时期，全市经济社会发展的主要目标是：主要经济指标增长速度不低于我市“十一五”以来平均水平、不低于东南沿海发展较快地区水平，在优化经济结构、壮大经济总量，深化改革、扩大开放，统筹城乡发展、建设宜居生态特大城市，提高城乡人民生活水平、实现安居乐业等方面取得新的突破，全力打造实力营口、活力营口、魅力营口、和谐营口。

　　到2015年，全市生产总值达到3000亿元，年均增长25%；地方财政一般预算收入达到300亿元，年均增长25%；全社会固定资产投资累计完成10000亿元，年均增长25%；社会消费品零售总额达到570亿元，年均增长18%；城镇居民人均可支配收入达到31000元，年均增长12%；农村居民人均纯收入达到15800元，年均增长13%。

　　“十二五”时期，全市经济社会发展的主要任务是：

　　——全面推进沿海经济带开发建设。按照“统筹规划，科学开发”的原则，以八大重点园区为载体，以项目建设为核心，着力打造百公里滨海生态城、百公里沿海产业带、百公里临海景观带，使之成为营口经济新的增长极。到2015年，沿海经济带生产总值达到2000亿元，年均增长27%；地方财政一般预算收入达到200亿元，年均增长42%；全社会固定资产投资累计达到8000亿元，年均增长30%。以沿海经济带开发建设为牵动，推动全市形成大开发、大开放新局面。积极申办产业基地出口加工区，争取营口港B型保税物流中心升级为保税港区。“十二五”时期，实际利用外商直接投资累计达到100亿美元，年均增长30%；外贸出口总额累计达到200亿美元，年均增长20%。

　　——做大做强工业经济。引导项目向园区集聚，推动产业向集群发展。着力打造钢铁及深加工、镁产品及深加工、石化、电机等四大千亿产业集群，着力打造输变电、船舶、港口机械、汽保设备、汽车配件等五大500亿产业集群，着力打造纺织服装、新材料、铝制品及深加工、工程塑料制品、乐器、印刷包装、管件阀门、食品饮品等八大百亿产业集群。大力发展新兴产业，努力在新能源、节能环保、海洋工程等领域实现新突破。创造更加宽松的环境，促进民营经济加快发展。到2015年，规模以上工业增加值实现1300亿元，年均增长20%；高新技术产业增加值实现390亿元，年均增长35%；民营经济增加值实现1000亿元，年均增长25%。

　　——大力发展城区经济和服务业。积极拓展城区发展空间，优化城区产业布局，着力打造温泉旅游、科技研发、辽河特色文化、金融、物流、商贸等六大服务业集聚区，努力扩大服务业规模。规划建设西炮台遗址公园等八大文化产业园，加快发展文化产业。到2015年，服务业增加值占生产总值比重达到41%以上，文化产业增加值占生产总值比重达到8%以上。站前区、西市区、老边区力争进入全省城区第一梯队，鲅鱼圈区进入全国城区先进行列。

　　——积极发展县域经济。抓好“三水一菜一牧”生产，加快农业集约化、标准化、品牌化步伐。盖州市要打造全国重要的苹果生产基地，大石桥市要建设优质稻米和南果梨生产基地。积极扶持发展农产品加工业，大石桥市、盖州市都要建立农产品加工园区。同时，深化农村综合改革，大力推进工业化和城镇化。大石桥市在全国百强县的位次要大幅度前移，盖州市要进入全国百强县行列。

　　——加快城市化进程。着力推进老城区、产业基地、北海新区、开发区、仙人岛五大城市组团建设。抓好北海新城等7个区镇和路南镇欢心甸村等6个村全省城镇化试点，加快虎庄镇等8个镇的小城镇建设。实施移民安居工程，努力扩大城市规模。到2015年，鲅鱼圈区、老边区实现全域城市化，其它市（县）区城市化水平要有较大提高。全市常住人口达到300万人，城市化率提高到75%。

　　——加强基础设施建设。推进营口港项目建设，力争港口吞吐量突破4亿吨，集装箱运量突破500万标箱。开工城际铁路、仙人岛疏港铁路、岫岩至熊岳高铁等项目，完成滨海景观路、产业基地至北海新区快速路等各组团之间连接路建设，实施庄林路外环线外移工程。加快营口机场建设，确保2013年通航。启动新一轮农村电网改造升级工程和华能仙人岛热电厂、华能大石桥热电厂等项目建设。加快推进海防堤、大伙房水库输水配套工程二期、头道河水库等水利工程。实施填海、造岛工程。加强信息化建设。

　　——提高自主创新能力。加快推进辽宁渤海科技城建设，努力打造科技研发总部基地。积极争取大专院校和科研院所在我市设立研发机构和合作基地，合作项目每年要超过百项。大力发展科技型企业，每年要有100个高科技项目开工建设。

　　——强化资源节约与生态环境保护。严格执行高耗能行业单位产品能耗限额标准，加大落后产能淘汰力度。大力发展循环经济，建设一批循环经济示范企业，实施一批节能减排项目。实施山体修复工程、河道治理工程，加强农村生态环境综合整治。到2015年，单位生产总值能耗降低15%，主要污染物排放减少10%，森林覆盖率提高到46.6%。

　　——提高城乡人民生活水平。实施山区富民工程，努力增加山区群众收入。深化收入分配制度改革，积极推进企业职工工资和离退休人员养老金总体水平与经济发展同步增长，最低工资标准高于全省平均水平。进一步完善覆盖城乡居民的社会保障体系，实现人人享有基本生活保障。大力促进就业再就业，城镇登记失业率控制在3.5%以内。

　　——加强社会建设。巩固基础教育“双高”水平，全面推进九年一贯制（寄宿制）学校建设，整合引进职业教育资源，建设1所以上本科大学，人均受教育年限达到11年以上。实施人才引进工程，引进各类人才总量年均增长20%以上。积极发展文化事业，提高医疗卫生服务水平，做好人口和计划生育工作，加快体育事业发展。实施“六五”普法，提高全民法律素质。加强社会治安综合治理，促进社会和谐稳定。

　　各位代表：“十二五”发展的宏伟蓝图已经绘就，尽管前进道路上面临着许多困难和挑战，但有省委、省政府和市委的正确领导，有市人大、市政协的监督支持，有全市广大干部群众的共同努力，规划确定的目标和任务一定会全面完成，一定能够为全市人民创造更多的福祉！

　　三、2011年重点工作安排

　　2011年是中国共产党成立90周年，也是“十二五”的开局之年。做好今年工作，确保“十二五”旗开得胜、首战告捷，事关今后5年目标的实现，事关营口振兴大业，任务艰巨，责任重大。

　　今年全市经济社会发展的总体要求是：按照“十二五”规划的总体思路，坚持以科学发展观为统领，紧紧围绕“抓升级，促转型，扩增量，惠民生”这一中心任务，千方百计保持经济发展的良好走势和增长动力，推进解决经济社会发展中的突出矛盾和深层次问题，确保经济高速增长、民生更加改善、社会和谐进步，实现“十二五”的高起点开局、高水平起步、高质量发展。

　　今年全市经济社会发展的主要预期目标是：地区生产总值完成1200亿元，增长20%；地方财政一般预算收入125亿元，增长25%；全社会固定资产投资1250亿元，增长25%；社会消费品零售总额290亿元，增长18%；城镇居民人均可支配收入19900元，增长12%；农村居民人均纯收入9700元，增长13%。在实际工作中，力争实现“两个不低于”的目标。

　　为实现上述目标，要重点抓好以下几项工作：

　　（一）着力抓好沿海经济带开发建设。举全市之力推进北海新区建设，全方位推进沿海开发开放，加快城市带、产业带、景观带的形成。

　　推进基础设施建设。加快仙人岛疏港铁路、深水航道、成品油及化工泊位等港口基础设施建设。营口港货物吞吐量达到2.5亿吨，集装箱运量达到380万标箱。北海新区要全面完成23平方公里启动区动迁和路网、水电气、公交站场等基础设施建设，加速推进具有鲜明特色的体育中心、会展中心、学校等功能性项目。开发区要进一步加大新区南移、工业北拓、城市东扩力度，高标准建设望海珍珠湾。大力推进产业基地二期及冶金化工重装备区、辽宁渤海科技城、仙人岛白沙湾滨海新城、中小企业园一期南扩区域和大石桥沿海新兴产业区基础设施建设，尽快达到项目入驻条件。

　　推进重点园区发展。北海新区要以打造“中国电机之都”为目标，积极培育壮大电机产业集群，引进电机产业项目累计达到500个。开发区要推进临港和滨海工业园区建设，着力打造辽宁沿海经济带科学发展示范区，开工千万元以上项目150个。产业基地要以发展港口机械产业集群为重点，加快大项目引进，开工千万元以上项目130个。积极争取产业基地晋升为国家级开发区。加快高新区建设，开工科技研发、新兴产业项目80个。仙人岛能源化工区要加快大型石化项目建设，积极培育千亿石化产业集群，开工千万元以上项目50个。中小企业园要开工千万元以上项目30个。大石桥沿海新兴产业区要开工千万元以上项目80个。以沿海经济带为重要载体，全力以赴扩大外贸出口。全市外贸出口总额达到26亿美元，增长20%。

　　推进体制机制创新。园区内设机构要勇于打破常规，按照效能优先的原则进行设置；要面向社会招聘领导干部和懂业务的专业人员，并做到能上能下、能进能出；建立重点园区开发建设情况通报制度，及时总结交流经验，鼓励先进、鞭策后进；完善园区领导班子绩效考评机制，激发园区发展活力。

　　（二）着力抓好招商引资。开展“项目年”活动，实施“1133”工程，即全市引进总投资千万元以上开工项目1000个，实际到位资金1200亿元，引进总投资5亿元以上开工项目300个，市直部门力争引进总投资千万元以上开工项目300个。全年引进内资650亿元，增长30%；实际利用外商直接投资12亿美元，增长40%。

　　完善招商机制。加强招商队伍建设，按照新机制运行。组织成立钢铁、石化、汽车、物流、机场、城际铁路等项目推进组，集中精力推进项目。严格领导包项目制度，实行“一把手”抓招商、谈项目。强化目标管理考核，对项目落地、资金到位及指标完成等情况实行月调度、季通报、年考核。

　　创新招商方式。坚持“走出去”、“请进来”相结合，深入开展主题概念招商和产业集群招商。组织好北海新区、辽宁渤海科技城和开发区船舶产业园、产业基地钢铁产业园、大石桥市镁质材料产业园、老边区汽保产业园等招商活动，确保签约一批项目。组织好赴香港、台湾和“长三角”、“珠三角”等地的大型招商活动，以及对日韩、欧美等重点区域和国内外500强大企业、大集团的招商，务求取得实效。

　　优化招商环境。完善绿色通道制度，精简审批环节，缩短审批时限，提高项目审批效率。对重大项目的签约、立项、开工、投产等实行全程跟踪落实的一条龙服务，确保洽谈项目早签约，签约项目早开工，开工项目早投产。加大舆论监督力度，促进软环境建设。加快信用营口建设，营造诚实守信的良好社会环境。

　　（三）着力抓好工业“五项工程”。以实施“五项工程”为重点，着力提升工业经济整体实力。规模以上工业增加值达到700亿元，增长20%。

　　实施企业提升工程。以提高企业资本运营能力、品牌影响力、核心竞争力为重点，加大对现有企业的扶持力度，促进企业产能和质量升级。继续实施中小企业成长工程，促进中小企业发展壮大。全市新增规模以上企业150户，新增销售收入超亿元企业80户，其中超10亿元企业5户。

　　实施产业集群工程。按照打造四大产值超千亿产业集群、五大产值超500亿产业集群、八大产值超百亿产业集群的要求，加快重大项目的引进和核心企业的培育，完善上下游关联企业的配套，拉长产业链条，加快产业集聚。

　　实施项目工程。实施大项目攻坚计划，全市引进世界500强或行业内世界100强投资项目2个以上；每个重点园区都要争取新上投资2亿元以上项目30个，其中5亿元左右项目10个，10亿元以上项目至少1个，确保一批重大项目开工建设。

　　实施并购工程。积极组织有条件的企业“走出去”，参与国内外市场竞争与资源配置，掌握并形成核心技术。完成并购国内外企业项目10个，引进海外研发团队8个。

　　实施节能降耗和淘汰落后产能工程。加大技术改造力度，促进企业向环保型、高科技方向迈进。积极发展清洁新能源、新材料、电子信息等低碳产业，确保完成省下达万元工业增加值能耗、用水量和钢铁、水泥等行业淘汰落后产能任务。

　　（四）着力抓好县域经济发展。全面启动新一轮县域经济发展“三年倍增”计划，县域生产总值、固定资产投资、地方财政一般预算收入分别增长27%、35%、30%。

　　毫不放松农业生产。既要抓好“米袋子”，又要抓好“菜篮子”。粮食产量达到70万吨，蔬菜产量达到95万吨，水果产量达到70万吨，水产品产量达到55万吨，肉蛋奶产量达到38万吨。大力发展规模化高效农业，新增专业乡10个、专业村50个，新建设施农业小区300个、1.5万亩。新增绿色和有机产品认证20个，新创盖州西瓜等省以上名牌15个。加大对农业的投入，搞好大型灌区改造、中小河流治理和气象减灾设施建设，积极推进农业机械化。

　　加快推进农业产业化。着力培育特色产业，盖州市、大石桥市按照新的农业定位，制定发展规划，完善推进措施，逐步形成生产规模。规范农民专业合作社发展，提高农民组织化程度。进一步发展农产品加工业，新增规模以上农业产业化龙头企业30个。加强农产品检验检测体系建设，完善政策性农业保险补贴政策。

　　加快推进工业化。以工业园区为载体，大力发展工业产业集群。加快大石桥市产值超千亿镁产品及深加工产业集群和产值超百亿铝制品及深加工产业集群、盖州市产值超500亿汽车配件产业集群和产值超百亿工程塑料制品产业集群建设。

　　加快推进城镇化。进一步完善县城发展规划，加快大石桥市城区南拓和盖州市城区西进，全力推进鲅鱼圈区、老边区全域城市化。重点抓好列入省规划的7个区镇和6个村的全省城镇化试点工作，务求早见成效、早出形象。

　　（五）着力抓好城区经济和服务业发展。启动新一轮发展城区经济“三年倍增”计划，调整区划拓展空间，下放权限激发活力，全市服务业增加值增长20%以上。

　　加快发展服务业集聚区和产业集群。重点抓好温泉旅游、科技研发等六大服务业集聚区建设。加快推进鲅鱼圈港前物流、仙人岛物流仓储、老边环渤海钢材城等物流园区建设，构建辐射东北的现代物流网络。引导和规范房地产市场，促进房地产业快速健康发展。鲅鱼圈区要重点抓好钢铁及深加工、输变电、船舶、纺织服装产业集群发展，站前区要重点抓好食品饮品产业集群发展，西市区要重点抓好管件阀门产业集群发展，老边区要重点抓好钢铁及深加工、汽保设备产业集群发展。

　　加快发展旅游业和文化产业。高标准制定和实施熊岳、双台温泉旅游区和鲅鱼圈海滨休闲旅游区发展规划，加快思拉堡温泉小镇、天沐海滨温泉城、奕丰温泉城、玉石文化博览园等重点项目建设，全面提高接待能力和服务水平。充分挖掘整合现有旅游资源，积极开发旅游产品，打造海滨温泉、历史遗迹、民俗文化等精品旅游景区和线路。加快金牛山古人类遗址、蟠龙山公园和迷镇山公园开发改造步伐。全年旅游总收入达到150亿元，增长30%。大力发展具有营口特色的文化产业，树立营口在全省乃至全国有影响力的文化品牌新形象。抓好辽河西大街开发建设和经营管理，努力打造中国历史文化名街。文化产业增加值实现30亿元，增长1倍。

　　努力扩大消费需求。继续落实积极的消费政策，发展消费信贷，改善居民消费预期，优化消费结构。努力提升居民消费能力，改善消费条件，培育新的消费热点。加强价格监管，保障市场供应，把居民消费价格涨幅控制在全省平均水平之下。

　　（六）着力抓好城市和生态环境建设。坚定不移地改造老城区，毫不动摇地建设新城区，做到老城有新变化，新城尽快出形象。

　　加强城市建设和管理。完成《营口市城市总体规划》修编，启动城市建设重点专项规划编制工作。开工建设滨海景观路、产业基地到北海的快速通道。加快营东新城、站前区“北方第一湾”、河海新西市“卧龙湾文化商贸区”建设步伐。推进营口机场建设，启动城际铁路建设。搞好铁路沿线环境综合整治，完成营口高铁站前广场建设。全面完成大伙房水库输水配套工程一期建设。加快推进输变电工程建设，做好华能营口热电二期前期工作。实施供暖、供水完善工程，加快供气设施建设。对主要街路实施绿化、亮化改造。加快垃圾处理厂建设，力争年底前投入使用。实施营柳河泵站新建工程，解决市区东部积水问题。完成东海大街排水工程建设。以推进城市数字化管理为契机，建立综合执法、小区物业和道路交通管理的长效机制。

　　加强生态环境建设。投资20亿元，实施造林绿化精品工程，完成造林绿化面积14.1万亩，新增城市绿地0.3万亩。启动山体修复工程、河道治理工程。加快实施农村环境连片整治工程，严格保护永远角湿地、山场林地、饮用水源地等重点区域。加快建设仙人岛展望公园、白沙湾浴场公园和浮渡河海上乐园等滨海景观。以世博“伦敦零碳馆”为牵动，推进低碳经济和低碳社区建设。

　　（七）着力抓好生产要素保障。下大气力破解资金、土地、人才三大瓶颈，为营口大发展、快发展提供有力保障。

　　加强金融支撑。集中精力利用好基金，全年引进基金100亿元；抓好企业上市工作，确保新增上市企业3家，力争达到5家；继续抓好引进金融机构工作，新开业银行业金融机构3家以上；组织一批融资贷款项目，全年新增金融机构贷款200亿元以上；继续发行企业债券，力争再发行20亿元。进一步加强担保机构建设，缓解中小企业融资难问题；积极探索BT、BOT、金融租赁等其它融资方式。

　　加强土地供给。抓住国家实施城乡建设用地增减挂钩试点的政策机遇，加快土地的整理和报批，积极争取“挂钩”指标；启动140平方公里填海造地工程，拓展土地利用空间；提高用地项目准入门槛，促进土地集约利用。

　　加强人才保障。实施人才引进工程，推广开发区经验，打造人才高地。启动移民安居工程，研究制定相关鼓励政策，吸引科技人才、管理人才和产业工人来营落户，力争引进移民5万人。加强与劳动力输出基地联系，积极开展用工洽谈等活动，千方百计组织与企业进行对接，切实解决企业用工短缺问题。

　　（八）着力抓好民生工程。改善民生是政府工作的出发点和落脚点，坚持工作重点向民生倾斜，资金重点向民生投放。

　　继续实施已启动的民生工程。全年实现实名制就业5万人，城镇登记失业率控制在3.5%以内；继续提高企业退休职工养老金标准、城乡低保标准、在乡复员军人生活补助标准、农村五保供养标准；稳定新型农村合作医疗保险参合率，积极推进实施国家基本药物制度综合改革试点工作；加快推进城乡社区建设。

　　启动一批群众关注、当年能够完成的民生工程。对北辰街、联盟路等12条主干道部分路段进行改造和建设；对金牛山大街、太和路等15条主干道道板进行改造；对辽河公园、镜湖公园等公园和建丰市场、花鸟鱼市场等市场进行改造；对70条街巷路进行综合改造；对60个旧住宅小区进行综合改造；新建廉租房3万平方米、经济适用房100万平方米，棚户区改造120万平方米；在主城区重点部位和居民集中居住区建设5个面积适中、设施齐全的市民休闲广场；在主城区建设5个地上多层停车场，增设重点路段隔离设施，建设交通诱导系统，对部分道路实行交通单向循环；在渤海大街和市府路交汇处进行综合改造，在东升市场建设3万平方米地下商业街，在金牛山大街和盼盼路交汇处建设2万平方米地下大型超市；新建、改扩建幼儿园各15所；完成100个村农民健身工程；解决农村10万人饮水安全问题；完成2万座农村改厕任务；启动山区富民工程，使东部山区161个贫困村年内人均纯收入增加500元；完成2个农村常年病人托管中心建设任务；新建1万平方米残疾人康复培训中心；实施计划生育特殊家庭关爱行动。

　　（九）着力抓好社会事业发展。坚持教育优先发展，完成营口大学园扩建二期工程，启动营口大学园新校区建设，新建营口职业技术学院，积极申办本科大学。统筹配置城乡教育资源，促进城乡教育均衡发展。积极发展文化事业，开工建设市博物馆、体验式文化广场，各市（县）区要至少建设一处综合文化活动中心。办好望儿山母亲节、海滨温泉节等节庆活动，丰富群众文化生活。加快推进广播影视数字化建设，为实现“三网融合”奠定基础。大力发展卫生事业，加快第一专科医院、第二人民医院建设，进一步推进医疗卫生体制改革，提高医疗卫生水平。认真做好人口和计划生育工作，稳定低生育水平。搞好健身中心等市民活动场所建设，广泛开展全民健身活动；发挥“一场三馆”作用，提高竞技体育水平。按照《全国文明城市测评体系》的标准要求，努力完成建设城市消防设施、设置城市避难场所等7项硬性指标和争创全国节水城市、国家卫生城市、全国充分就业城市3项特色指标，争创全国文明城市。

　　启动“六五”普法，抓好社会稳定、安全生产和食品药品安全工作。加强国防后备力量建设，积极开展双拥共建活动，创建全国双拥模范城。进一步做好妇女儿童、民族宗教、外事侨务、气象、地震、档案、史志、人防、老龄、残疾人、红十字等项工作。

　　（十）着力抓好政府自身建设。加强民主法制建设，自觉接受人大及其常委会的法律监督和工作监督，自觉接受政协的民主监督。认真办理人大代表的建议、批评、意见和政协提案，不断提高满意率和落实率。高度重视新闻舆论监督和社会公众监督，充分保障人民的知情权、参与权、表达权、监督权。按照“为民、务实、清廉”的要求，努力建设人民满意的政府。

　　加强能力建设。在深入学习上下功夫，不断提高政府班子和政府组成人员领导科学发展的能力、驾驭全局的能力、依法行政的能力和调处各种复杂矛盾的能力。加强公务员队伍建设，努力造就一支肯钻研、懂业务、有能力、干事业的公务员队伍。不断提高政府工作的创造力、决策力和执行力，切实解决重点、难点、热点问题。

　　加强作风建设。始终保持奋发有为的精神状态，充满发展激情；坚持实事求是、真抓实干，做到摸实情、出实招、办实事、求实效；坚持走进矛盾、面对问题，把一个项目一个项目抓实，把一件事一件事办好；坚持雷厉风行、提高效率，做到定了就办，办就快办，办就办好；坚持主动服务、超前服务、创新服务，营造良好的发展环境。

　　加强廉政建设。按照廉政建设要求，进一步完善惩治和预防腐败体系。规范权力运行，从源头上预防腐败发生。强化对重大工程、重点行业、重点领域和重要环节的管理，坚决纠正损害群众利益的不正之风。坚决进行反腐败斗争，决不让腐败问题影响营口的整体形象、决不让腐败问题影响营口的发展大业。

　　各位代表：创造新业绩，实现新跨越，建设新营口，这是全市人民的殷切希望。市政府将在省委、省政府和市委的正确领导下，在市人大、市政协的监督支持下，以更加昂扬的斗志、更加饱满的激情，开拓创新、扎实工作，努力完成历史赋予的各项任务，再创营口振兴发展的新辉煌！
